# Effectiveness of a cognitive-behavioral group intervention for knee osteoarthritis pain: protocol of a randomized controlled trial

**DOI:** 10.1186/1471-2474-14-46

**Published:** 2013-01-29

**Authors:** Eeva-Eerika Helminen, Sanna H Sinikallio, Anna L Valjakka, Rauni H Väisänen-Rouvali, Jari P Arokoski

**Affiliations:** 1 Department of Physical and Rehabilitation Medicine, Kuopio University Hospital, P.O.B. 1777, Kuopio, FI-70211, Finland; 2 Kuopio Health Centre, P.O.B. 227, Kuopio, FI-70101, Finland; 3 Institution of Public Health and Clinical Nutrition, University of Eastern Finland, Kuopio Campus, P.O.B. 1627, Kuopio, FI-70211, Finland; 4 Substance Abuse and Mental Health Unit of Raisio, Nallinkatu 3, Raisio, FI-21200, Finland; 5 Institute of Clinical Medicine, University of Eastern Finland, Kuopio Campus, P.O.B. 1627, Kuopio, FI-70211, Finland

**Keywords:** Osteoarthritis, Pain management, Cognitive-behavioral, Psychological, Intervention study

## Abstract

**Background:**

Knee osteoarthritis is the most common type of arthritis, with pain being its most common symptom. Little is known about the psychological aspects of knee osteoarthritis pain. There is an emerging consensus among osteoarthritis specialists about the importance of addressing not only biological but also psychosocial factors in the assessment and treatment of osteoarthritis. As few studies have evaluated the effect of psychological interventions on knee osteoarthritis pain, good quality randomized controlled trials are needed to determine their effectiveness.

**Methods/Design:**

We intend to conduct a 6-week single-blinded randomized controlled trial with a 12-month follow-up. Altogether, 108 patients aged from 35 to 75 years with clinical symptoms and radiographic grading (KL 2–4) of knee osteoarthritis will be included. The clinical inclusion criteria are pain within the last year in or around the knee occurring on most days for at least one month, and knee pain of ≥40 mm on a 100-mm visual analogue scale in the WOMAC pain subscale for one week prior to study entry. Patients with any severe psychiatric disorder, other back or lower limb pain symptoms more aggravating than knee pain, or previous or planned lower extremity joint surgery will be excluded. The patients will be randomly assigned to a combined GP care and cognitive-behavioral intervention group (n = 54) or to a GP care control group (n = 54). The cognitive-behavioral intervention will consist of 6 weekly group sessions supervised by a psychologist and a physiotherapist experienced in the treatment of pain. The main goals of the intervention are to reduce maladaptive pain coping and to increase the self-management of pain and disability. The follow-up-points will be arranged at 3 and 12 months. The primary outcome measure will be the WOMAC pain subscale. Secondary outcome measures will include self-reports of pain and physical function, a health related quality of life questionnaire, and various psychological questionnaires. Personnel responsible of the data analysis will be blinded.

**Discussion:**

This study addresses the current topic of non-pharmacological conservative treatment of knee OA-related pain. We anticipate that these results will provide important new insights to the current care recommendations.

**Trial registration:**

Current Controlled Trials ISRCTN64794760

## Background

Osteoarthritis (OA) is the most common form of arthritis and a major contributor to functional disability [1]. It represents a major social and health problem in the elderly, imposing an increasingly heavy economic burden on social welfare and health care systems. This is due to the need for surgical and medical interventions and frequent absenteeism from work [2]. Although not all OA is symptomatic, the World Health Organization has estimated that OA is the cause of disability in at least 10% of the population over the age of 60 years [3].

Knee OA is the most common type of arthritis. The main symptom of knee OA is pain, which is generally related to joint use and relieved by rest. However, the association between radiological changes in knee OA and the severity of pain or the level of disability is not straightforward. As OA progresses, the pain may become more persistent and also manifest itself at rest. In addition to pain, loss of function and joint stiffness are typical symptoms of OA, which often lead to difficulties in performing daily activities.

The assessment and treatment of pain is vital to the management of OA [4]. It is known that chronic pain is associated with increased considerable psychological distress, such as anxiety and depression. One population study from 17 different countries found that depression and anxiety disorders occurred significantly more often in individuals with self-reported arthritis, with depression present in 5−10% of those with arthritis [5]. In another study conducted by Smith and Zautra, measures of anxiety and depression emerged as independent and significant predictors of current and future pain [6].

The cognitive-behavioral (CB) perspective presented by Turk et al. [7] is the most widely accepted model in the field of pain psychology. It has led to the identification of cognitive and other psychological factors that are associated with pain severity and disability. If one considers the constructs that have the strongest empirical support, factors like pain catastrophizing [8], fear-avoidance [9], self-efficacy and lack of perceived control [10,11], and passive pain coping [12] have been claimed to be of importance. Factors, such as pain catastrophizing and pain-related fear, have been found to be strongly and consistently associated with pain severity and disability in patients with musculoskeletal pain [13] and knee OA [4]. Low self-efficacy and helplessness have also been identified as predictors of disability in OA patients [14,15].

According to several reviews, psychological factors influence not only pain and disability, but also in particular the transition from the presence of acute to chronic pain [13,16]. However, there have been relatively few attempts to prevent chronic disability in OA by adopting a CB approach. Calfas et al. conducted a randomized controlled trial (RCT) testing a 10-week group-based CB program on 40 OA patients [17]. They concluded that in the long term, physical and psychological functioning did not differ between the CB and the education control group. In 2011, Riddle et al. published the results of a quasi-experimental study testing an 8-session individual pain coping skills training (PCST) program in patients with elevated pain catastrophizing who were scheduled for knee arthroplasty [18]. They found that the PCST program resulted in significantly greater reductions in pain severity and catastrophizing, and greater improvements in function when compared with the usual care cohort. Somers et al. recently reported the results of an RCT studying the effects of a PCST program and a behavioral weight management intervention [19]. They concluded that the combination of these two treatments yielded significantly better outcomes in terms of pain, physical disability, stiffness, activity, arthritis self-efficacy, and weight self-efficacy than either of the intervention modalities alone or that evident in the control group.

There are several trials that have used behavioral interventions with similarities to CB principles as well as studies that have integrated CB principles with other forms of rehabilitation in OA patients. For example, Hurley et al. conducted an RCT assessing the effects of a combined exercise, self-management, and active coping strategies rehabilitation program [20]. They found over the long-term, that the rehabilitated participants enjoyed better physical function, lower community-based health care costs, medication costs, and total health and social care costs as well as concluding that there was a high probability (80–100%) that the program was cost effective. On the other hand, Keefe et al., adopted an RCT setting to investigate the separate and combined effects of spouse-assisted pain coping skills training and exercise training in patients having persistent osteoarthritic knee pain [21]. They concluded that the kind of intervention combining spouse-assisted coping skills training and exercise training could improve physical fitness, strength, pain coping, and self-efficacy in patients suffering from pain due to OA.

One CB-based approach in treating pain-related disability and chronicity is the 6-session group intervention model presented by Linton [22]. The model was originally developed for early identification and intervention in the prevention of musculoskeletal pain [23,24]. The standardized 6-session program focused on coping, function and cognitions and the application of learning principles to allow the individual to utilize more adaptive methods of pain management and active coping [22]. The model has previously been tested in RCT setting in patients with back and neck pain [23-25]. In these studies, the CB intervention led to less short- and long-term work absenteeism, fewer health care visits, decreased perceived risk and fear-avoidance beliefs, and a larger number of pain-free days.

The present study aims to explore the effectiveness and cost-effectiveness of the CB group intervention described by Linton [22], modified for patients with knee OA. As far as we are aware, there have been no previous studies using this approach in patients with knee OA. The working hypothesis is that patients with symptomatic knee OA will benefit from this kind of CB rehabilitation program. More specifically, we intend to examine the effect of the intervention in terms of self-reported physical function and pain, pain-related work abstinence, the number of pain-related health care visits, and health-related quality of life (HRQoL). We also aim to determine the effect of the intervention on several psychological variables such as depression, anxiety, sense of coherence, pain catastrophizing, kinesiophobia, self-efficacy, and life satisfaction. Finally, we plan to run a cost-utility analysis of the intervention based on quality-adjusted life years (QALY).

## Methods/Design

### Design

**Figure 1:**
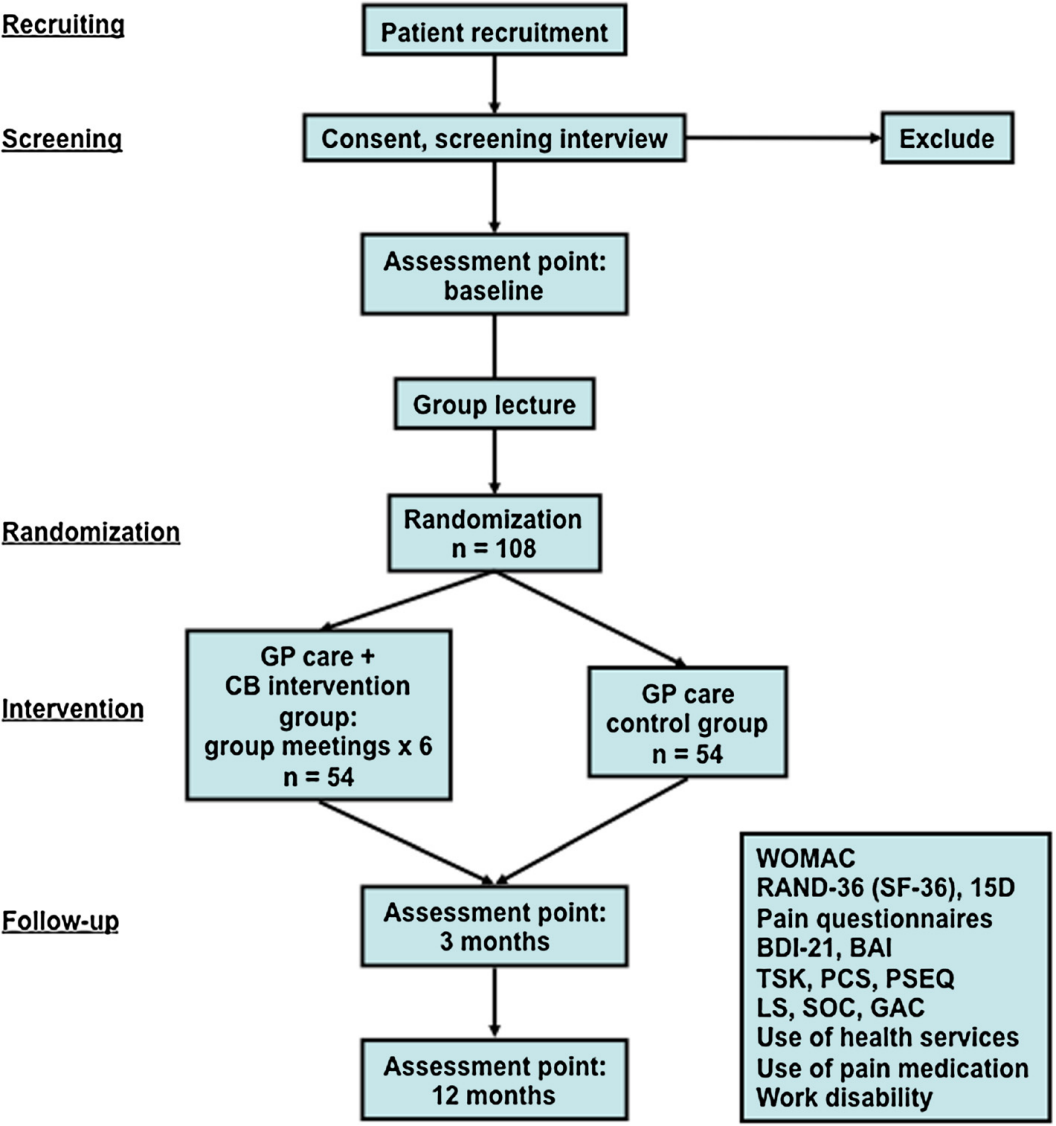
**The study design.** GP = general practitioner; CB = cognitive-behavioral; WOMAC = Western Ontario and McMaster Osteoarthritis Index; RAND-36 = the RAND 36-item health survey; 15D = generic 15D instrument; BDI-21 = 21-item Beck Depression Inventory; BAI = Beck Anxiety Index; TSK = Tampa Scale for Kinesiophobia; PCS = Pain Catastrophizing Scale; PSEQ = Pain Self-Efficacy Questionnaire; LS = life satisfaction; SOC = sense of coherence; GAC = global assessment of change.

The proposed research is a 6-week open study with follow-up-points at 3 and 12 months from the beginning of the study. The participants will take part in 6 group meetings each lasting 2 hours. The meetings will be supervised by a trained psychologist and a physiotherapist according to a CB intervention model presented by Linton [22]. At the beginning of the study, prior to the randomization, all the patients will participate in groups of about 20 people to hear a lecture regarding the current treatment guidelines for knee OA provided by the study doctors. They will also receive the patient-version booklet of the Finnish Current Care (CC) guideline on knee and hip OA [26]. The study protocol does not interfere with the usual care that the participants may receive from their general practitioner (GP) in primary health care during the study period. Questionnaires will be sent to all participants at the beginning of the study and at two follow-up points (3 and 12 months). The inclusion and exclusion criteria for the study patients are listed in Table 1. The study design is illustrated in Figure 1.

**Table 1:** Inclusion and exclusion criteria

**Inclusion criteria:**	
1.	Age 35–75 years
2.	Pain within the last year in or around the knee occurring on most days for at least a month [27]
3.	Knee pain greater than or equal to 40 mm on a 100-mm visual analogue scale (VAS) in the WOMAC* [28-31] pain subscale for one week prior to study entry
4.	KL** 2–4 [32] radiographic knee osteoarthritis
5.	Able to attend 6 intervention sessions
**Exclusion criteria:**	
1.	Severe psychiatric or psychological disorder***
2.	Other back or lower limb pain symptoms more aggravating than knee pain
3.	Previous or planned lower extremity joint surgery
4.	Inability to complete the study ****

*Western Ontario and McMaster Universities Osteoarthritis Index.

**Kellgren–Lawrence radiologic score for knee osteoarthritis.

***Psychotic illnesses or psychological disorders that had led to hospitalization or disability to work.

**** Inability to fill in the questionnaires or uncertainty in ability to complete the study due to possible changes in the near future related to health, family, or living conditions.

### Participants and recruitment

Our aim is to recruit 108 patients mainly from primary care providers in the Kuopio area of eastern Finland. Our primary recruitment strategy is to dispatch recruitment letters to patients aged from 35 to 75 years who have had knee radiographs with knee OA changes taken in public primary care locations (Kuopio Health Centre or Kallaveden Työterveys). The study doctors will check the knee radiographs of each patient from an X-ray database and knee radiographs will be graded according to the Kellgren-Lawrence (KL) classification [32]. The recruitment letter will be sent to those subjects with KL grade ≥2, which has been used as a cut-off to classify knee OA [33]. In conjunction with this recruitment strategy, advertisements requesting potential study candidates to contact the study doctors will be placed in the facilities of local primary care providers as well as in the outpatient clinics of the Department of Orthopedics and the Department of Physical and Rehabilitation Medicine at Kuopio University Hospital. Physicians and surgeons working at these locations will also be informed about the study and asked to distribute the advertisements to patients with knee OA.

All study candidates will receive a recruitment letter containing information about the study as well as a preliminary questionnaire and an informed consent form. The preliminary questionnaire will contain a comprehensive list of other comorbidities, including psychiatric illnesses. In the preliminary questionnaire the patients will be asked if they have other back or lower limb pain symptoms that are more aggravating than their knee pain (yes/no). After receiving the signed informed consent and the questionnaire, the study doctors will check the knee radiographs based on KL classification and determine the patient’s eligibility according to the inclusion and exclusion criteria of the study (Table 1). Potential study patients will be able to contact the study doctors by phone or email throughout the recruitment process with any questions concerning the trial.

The patients will be randomly assigned to either the GP care and intervention group (n = 54) or the GP care control group (n = 54). Randomization will be conducted in blocks of six, separately for men and women. A computer-generated code for randomization will be constructed by a statistician who will not meet the study patients, and it will be administered via sealed opaque envelopes. The personnel responsible for data collection will be blinded to group assignment and will not be involved in providing the interventions. The code for randomization will be opened only after the statistical analyses have been performed after the 12-month follow-up point.

### Intervention

A CB group intervention with 6 weekly sessions will be supervised by an experienced psychologist and a physiotherapist. The sessions will take place in a group of 8–10 persons according to the model presented by Linton [22]. Each session will last for 2 hours with a 15–20-minute break to enhance peer support and social bonding. The outline of the sessions will include an introduction (15 min), lecture (knowledge and insight, max 15 min), problem solving (in pairs/teams, 15–20 min), skills training (15–20 min), homework assignments (15 min) and a résumé (feedback) of the session (15 min). A patient example of a knee OA pain patient will be used throughout the intervention as a basis for discussion and practice in problem solving. An outline of each session is presented in Table 2. The psychologist is the principal leader of the CB intervention. The physiotherapist’s tasks are to lead the relaxation exercises, provide the information of OA pain mechanisms in the first session, offer advice about suitable exercises in the second session, and facilitate the group in general when needed. Both the intervention and the control group will continue side by side with the usual GP care that patients may receive in the primary care throughout the study.

**Table 2:** **An overview of the content of the cognitive-behavioral intervention based on Linton (2005) **[22]

**Session**	**Focus**	**Skills**	**Objectives**
1	Causes of pain and the prevention of chronic problems	Problem solving	•To provide information about the causes of pain.
		Applied relaxation	•To provide information about the risk of chronic pain problems.
		Learning and pain	•To help participants in identifying relevant factors in one’s own pain problem.
			•To train problem-solving and relaxation skills.
			•To teach pain control techniques.
2	Managing your pain	Activities, maintain daily routines	•To provide information about the relationship between activity and musculoskeletal pain.
			Scheduling activities	
		Relaxation training	•To help participants in understanding fear avoidance behavior.	
			•To teach participants to identify goals for a satisfying activity level.	
			•To teach management skills: scheduling, pacing, graded increase.	
			•To teach cognitive skills to minimize problems with activities.	
			•To introduce stress and stress management.	
3	Promoting good health, controlling stress at home and at work	Warning signals	•To provide information how pain problems may be prevented.	
		Cognitive appraisal	•To provide information how to utilize thoughts and behaviors in preventive efforts.	
		Beliefs	•To teach how to apply various skills (relaxation, activity management, beliefs, pauses etc.) as coping.	
			•To help the participants to identify targets for developing coping strategies.	
			•To teach applied relaxation as coping strategies.	
4	Adapting for leisure and work	Communication skills	•To provide opportunities to receive reinforcement for correct ”coping” approximations from the group.	
		Assertiveness		
		Risk situations	•To provide information about how workplace and family may be influenced by the participant’s pain problem.	
		Applying relaxation		
			•To provide information and coping strategies concerning situations where the workplace and family may influence the participant’s pain perceptions.	
			•To teach assertiveness in using the coping skills learnt.	
			•To help participants to identify supportive behaviors from others.	
			•To teach participants to prompt these behaviors to promote positive relationships with family and friends.	
			•To teach how to apply rapid relaxation to risk situations.	
			•To teach participants how to employ several coping techniques in social situations.	
			•To begin to plan a personal coping program.	
5	Controlling flare-ups	Plan for coping and flare-ups	•To provide information about flare-ups and maintenance.	
		Coping skills review	•To teach how to use applied relaxation as coping.	
		Applied relaxation	•To teach how to apply their skills to cope with flare-ups.	
		Own program	•To develop a personalized coping program.	
			•To develop a self-care strategy that may reduce the need for healthcare visits.	
6	Maintaining and improving results	Risk analysis	•To reinforce appropriate coping behaviors.	
		Plan for adherence	•To provide information about maintenance and adherence.	
		Own program finalized	•To teach participants to do risk analysis and enhance adherence.	
			•To teach participants about enhancing and fine-tuning their program.	
			•To evaluate the course and participants’ progress.	

The same psychologist and physiotherapist who are both experienced in group-based rehabilitation interventions as well as in pain management will arrange the CB program for all the intervention groups. They have been trained for this particular CB intervention by going through in detail the session manual for therapists [22] and its Finnish translated version together with the other members of the research group.

### Outcome assessment

The assessment points of this study are the baseline and 3 and 12 months, at which time points the questionnaires will be sent by post to the patients with a pre-paid return envelope. At the baseline, information will be gathered on the demographics, comorbidities, work history, and previous rehabilitation measures. The primary and secondary outcomes and other measures are listed in Table 3.

**Table 3:** Outcomes and other measures

Primary outcome measure*	
Self-reported pain	WOMAC (VAS) [28-31] pain subscale
Secondary outcome measure*	**Measurement**
Self-reported physical function, pain and stiffness	WOMAC (VAS) physical function and stiffness subscales [28-31], NPRS, mean and worst pain (past week, 3 months)
Depression, anxiety, sense of coherence, pain catastrophizing, kinesiophobia, self-efficacy, and life satisfaction	BDI-21 [34-37], BAI [38,39], 13-item SOC scale [40-46], PCS [47,48], TSK [49,50], PSEQ [51,52], 4-item LS scale [53-55]
Health-related quality of life and cost effectiveness	RAND-36 (SF-36) [56,57], 15D [58], QALY [58], OA-related sick leave, use of pain medication, knee OA-related health care visits, rehabilitation and pensions
GAC	GAC
**Other measures**	**Measurement**
Identifying risk for persistent pain	Örebro MPQ [59]
Major life events	Open question
Adherence	Attendance at meetings

*The primary end point for data analysis is 12 months. All outcome measures will be undertaken at baseline and after 3 and 12 months. WOMAC = Western Ontario and McMaster Osteoarthritis Index; NPRS = numeric pain rating scale; BDI-21 = 21-item Beck Depression Inventory; BAI = Beck Anxiety Index; SOC = sense of coherence; PCS = Pain Catastrophizing Scale; TSK = Tampa Scale for Kinesiophobia; PSEQ = Pain Self-Efficacy Questionnaire; LS = life satisfaction; RAND-36 = the RAND 36-item health survey; 15D = generic 15D instrument; QALY = quality-adjusted life years; OA = osteoarthritis; GAC = global assessment of change; MPQ = Musculoskeletal Pain Questionnaire.

The primary outcome measure of the study is the Western Ontario and McMaster Universities Osteoarthritis Index (WOMAC) self-reported pain subscale [28,29], which is to be measured with the pain subscale of the Finnish version of the WOMAC [30,31]. The Finnish WOMAC consists of three dimensions: pain (5 items), stiffness (2 items), and physical functioning (17 items). Responses for the 24 items are registered on a 0–100 mm visual analogue scale (VAS).

Secondary outcomes of the study will include WOMAC stiffness and physical function subscales [28-31], as well as numeric pain rating scales of the worst and mean pain during the previous week and past three months. The health-related quality of life will be evaluated with 15D [58] and RAND-36 [56,57]. The use of pain medication, the number of knee OA-related health care visits, sick leave days, rehabilitation and pensions will be recorded. The patients will also be asked to complete several psychological questionnaires at the assessment points: depression will be evaluated with Beck's Depression Inventory (BDI-21) [34-37], anxiety with Beck's Anxiety Index (BAI) [38,39], sense of coherence with a 13-item sense of coherence scale (SOC) [40-46], catastrophizing with the Pain Catastrophizing Scale (PCS) [47,48], kinesiophobia with Tampa Scale of Kinesiophobia (TSK) [49,50], pain self-efficacy with Pain Self-Efficacy Questionnaire (PSEQ) [51,52], and life satisfaction (LS) with a 4-item LS scale [53-55]. Finally, some questions concerning major life events and the global assessment of change will be included.

### Statistical analysis

The mean (±SD) knee joint pain score (WOMAC, VAS) was estimated by using the results of the knee joint pain scores in previous studies [60,61]. Since the knee pain has to be ≥40 mm on a 100-mm VAS (WOMAC) in this present study, we postulated that there will be a mean of at least 48±16.2 mm in the WOMAC pain subscale at baseline. In our study the 20% reduction in primary outcome (WOMAC pain) due to the intervention was considered as being clinically relevant in accordance with the OMERACT-OARSI set of responder criteria [62]. In the comparison of the mean pain scores between the groups, 54 patients per group are needed according to a power calculation with the two-tailed Student t-test with a 5% significance level and 80% power, assuming a 20% dropout rate [63].

Demographic characteristics and baseline data will be summarized by descriptive statistics. Randomly missing data in the longitudinal set-up will be imputed using expectation–maximization algorithm before the analysis in order to follow the intention to treat principle. Data on various psychological variables predicting reported knee pain will be assessed by multiple regression analysis. For outcomes measured on a continuous scale, differences between groups in the mean change from the baseline to 12 months will be evaluated using the linear mixed modeling. The model assumptions will be checked by standard diagnostic plots. The participant rating of the global assessment of change will be compared between the two groups using the two-tailed Student t-test.

Cost-effectiveness of the intervention will be evaluated by cost-utility analysis which is a techniques that incorporates the expenses of the intervention as well as the costs of the use of health care services and pain medication, sick leaves, rehabilitation and pensions. Utility analysis is based on measurement of QALY from 15D [58]. Life expectancy with 0%, 3% and 5% discounting will be incorporated in the analysis. The cost-effectiveness of the intervention can be evaluated by dividing the overall costs with the QALY.

## Discussion

There are several reasons why this study will advance our understanding of effective interventions to improve conservative knee OA treatment. First, there have only been a few studies evaluating the effect of psychological interventions on knee OA pain. Thus, good quality RCTs are needed to determine their effectiveness. As present, very few studies have evaluated a CB intervention for knee pain treatment, as far as we are aware this will be the first study to tackle this particular CB intervention model in treating OA patients. Second, the proposed research will address the very current topic of non-pharmacological conservative treatment of knee OA, and the results may have a significant impact on current care recommendations. Since knee OA is the most common form of arthritis, representing a major economic burden on social welfare and healthcare systems for both society and individual patients, it is worthwhile to find novel cost-effective interventions promoting conservative treatment. Third, it is important to evaluate this kind of CB intervention since cognitive and psychological factors are known to play major roles in chronic pain, pain severity and disability. Moreover, evidence-based guidelines indicate that one should adopt a the combination of pharmacologic and nonpharmacologic modalities to achieve adequate management of knee OA [64-66]. With respect to nonpharmacologic treatment modalities, it has been occasionally recommended that patients should participate in self-management programs with psychosocial interventions [64]. We hope that the results of the cost-effectiveness and cost-utility analysis of the proposed study will favour their inclusion into the current care recommendations in the future.

The study protocol has some limitations. First, due to volunteer bias, the results of psychological intervention studies may have limited generalizability [67]. Second, as the majority of the study patients will probably originate from primary care, the results of this study will mainly apply to that environment. Third, the CB protocol presented by Linton [22] indicates that patients participating in a CB-type intervention should be evaluated to ensure that they are at risk of developing a persistent pain problem. Linton himself has developed a questionnaire, the Örebro Musculoskeletal Pain Questionnaire (MPQ) [59], to be used for this purpose. In our study, however, we have decided not use this questionnaire or any other cognitive or psychological evaluation as part of the inclusion criteria. One reason for this decision is that the Örebro MPQ has only been validated in back pain patients, and there is a lack of data concerning the similarity of pain experiences in back and knee OA pain in general. Furthermore, our inclusion criteria already include several pain measures, assessing both intensity and duration of pain. We consider that by applying to these criteria, in all probability, the patients would be at risk of developing a persistent pain problem if they were not already suffering from it. However, the Örebro MPQ will be included in the baseline patient questionnaire for analysis purposes. With the availability of the final data of the study, it will be interesting to see whether those patients determined to be at risk of developing a persistent pain problem according to the Örebro MPQ have benefited more from this intervention.

Another limitation of the study might be the different amount of attention paid to the two groups. As the group of patients in the intervention will receive 12 hours more attention, a Hawthorne effect [68-70] is probable. However, all patients will receive the same basic instructions about knee OA treatment according to Finnish CC guideline [26] when they are listening to a lecture delivered by a general practitioner prior to the randomization. This lecture will be held in groups of about 20 people and the whole research group will be present to answer questions. During the lecture the patient-version booklet of Finnish CC guideline on knee and hip OA [26] will be handed out to all participants. The participants in both groups also will continue to receive standard care (i.e. normal routine care offered by their own general practitioners including analgesics and physiotherapy).

BDI-21, BAI, 13-item SOC scale, TSK, PSEQ and LS questionnaires have been used in several clinical studies in Finland [35-37,39,44-46,50,52-55]. However, at present BDI-21 and 13-item SOC scales have been validated in Finland [37,46] and the validation process of TSK is almost complete. We have available translated versions of separate questionnaires that have been in use in clinical studies and clinical routine in Finland. As the psychometric questionnaires are all secondary outcome measures in our study we decided to include them even though there is a shortage of supporting evidence for the validity of some of their psychometric measurement properties in Finnish patients.

Obtaining and assessing KL grades are not simple tasks. Valid measures are dependent on knee flexed standing views [71,72] and examiner experience [73,74]. Although it has recognized weaknesses, the KL score has been the most widely used radiographic score to assess knee OA [73]. We decided to use KL grade ≥2, which has been extensively used as the inclusion criterion in knee OA studies [33]. Unfortunately, we will not be able to control the radiographic quality since the knee X-rays will have been taken in public primary care locations and in the outpatient clinics. However, we use the combined radiographic and clinical criteria that have been proposed for use when diagnosing knee OA. If one combines the clinical and radiographic factors, the sensitivity and specificity of knee OA diagnosis are 91% and 86%, respectively [75]. The observers also will be well trained and acquainted with the radiographic atlas of the KL scores before the start any assessments. Although reliability for the separate KL radiographic features may depend on the level of the investigator’s experience, the intra- and inter-observer reliability for the overall score is known to be relatively high [74].

No significant side effects or adverse events are expected.

## Competing interests

The authors declare that they have no competing interests.

## Authors’ contributions

E-EH, JA and SS designed the trial protocol. E-EH and JA will be responsible for patient recruitment. SS, RV-R, E-EH and JA adjusted the intervention program to OA patients. SS and RV-R will hold the intervention sessions for the participants. E-EH, JA and SS drafted the manuscript. AV translated the Session manual for therapists. E-EH translated the patient example of the Session manual for therapists. JA and E-EH applied for the project funding. All authors have read and approved the final manuscript.

## Pre-publication history

The pre-publication history for this paper can be accessed here:

http://www.biomedcentral.com/1471-2474/14/46/prepub
